# Advancing feminist understandings of woman abuse: the value of old wine in new bottles

**DOI:** 10.3389/fsoc.2025.1550645

**Published:** 2025-02-11

**Authors:** Walter S. DeKeseredy

**Affiliations:** Department of Sociology and Anthropology, West Virginia University, Morgantown, WV, United States

**Keywords:** gender, research methods, patriarchy, feminism, sociology

## Abstract

Despite making some of the most important advances in the social scientific study of woman abuse, feminist sociological research and theorizing that prioritize the concept of patriarchy have leveled off or declined in the last 15 years due, in large part, to the current hegemony of mainstream criminologists fundamentally opposed to a rich gendered understanding of one of the world's most compelling social problems. Drawing on nearly 50 years of research done by an international cadre of highly experienced scholars, this article demonstrates the importance of revisiting some major feminist conceptual, empirical, and theoretical contributions from the past. Recommended here are gender-specific conceptualizations of abuse, in-depth reviews of the extant literature, self-report surveys of potential male offenders, quantitative indicators of men's patriarchal attitudes and beliefs, and supplementary open-ended survey questions.

## Introduction

It is an understatement to declare that empirical and theoretical social scientific work on various types of woman abuse in private and public places has mushroomed over the past 50 years. Standing back to view these extraordinary achievements, one can only be awed at the coalition of feminist scholars who did so much in so little time. Still, regardless of making some of the most important advances in the field, feminist sociological research and theorizing that prioritize the concept of patriarchy have leveled off or declined in the last 15 years due, in large part, to the current hegemony of mainstream scholars fundamentally opposed to a rich gendered understanding of one of the world's most compelling social problems (Pease, 2019; DeKeseredy et al., 2023; Ip et al., 2024). Drawing on five decades of research and data generated by an international cadre of highly experienced scholars, this article demonstrates the importance of revisiting some major feminist conceptual, empirical, and theoretical contributions from the past. Recommended here are gender-specific conceptualizations of abuse, in-depth reviews of the extant literature, self-report surveys of potential male offenders, quantitative indicators of men's patriarchal attitudes and beliefs, and supplementary open-ended survey questions. It is first necessary, however, to account for the current marginalization of feminist understandings of woman abuse.

## The weakening of feminist inquiry: what happened?

Some of the most important critiques of feminist inquiry of any sort have come from debates among feminists that are designed to advance social scientific ways of knowing (DeKeseredy, 2022; Miller, 2003). Donald Trump and his followers, on the other hand, are strongly committed to maintaining sexist gender roles, norms, and beliefs, which hinders productive discussions about why patriarchy persists and how it can be addressed. Trumpists, too, are fueling a long-standing antifeminist backlash, especially in the U.S. (Chesney-Lind, 2019; DeKeseredy, 2020; Rothe and Collins, 2020). What Dragiewicz (2018, p. 334) stated 7 years ago still holds, and her description of the political, economic, and cultural context at that time is destined to get much worse:

Trump took power in the context of overtly sexist commentary and revelations of an alarming catalog of candidates' and appointees' personal histories of violence against women. Once in office, the Trump administration immediately began to shore up structural discrimination via attacks on women's reproductive rights, health care for pregnant women, implementing new forms of racialized discrimination against immigrants, and support for failed “tough on crime” policies. Rolling back the clock on incremental gains made over decades, the administration undertook wholesale destruction of programs and institutions intended to ameliorate the harms of equality.

Rigorous feminist empirical and theoretical work on woman abuse, which will be defined in the next section of this article, did not wither in the past 15 years solely because of Trumpism. Patriarchy is an “age-old structure” born long before Trump became President (Gilligan and Snider, 2018), and men have been physically, sexually, psychologically, and economically abusing women for centuries (Dobash and Dobash, 1999; DeKeseredy and Donnermeyer, 2023). Miller (2017, p. 3) reminds us:

Patriarchy… as embedded in the Old and New Testaments in the Bible and Roman legal precepts, has been a powerful organizing concept with which social order has been understood, maintained, enforced, contested, adjudicated, and dreamt about over two millennia in Western History.

Related to this point is the fact that “the force and furor of the backlash” always churn “beneath the surface, largely invisible to the public eye” (Faludi, 1991, p. xxi). Instead of being a new problem for feminists, the backlash is a “recurring feature in the history of feminism. Feminist successes have often been met, not only with resistance, but with renewed determination by patriarchal forces to maintain and increase the subordination of women” (Walby, 1993, p. 79). Nonetheless, it would be an oversight not to mention that feminist perspectives that critically examine male-to-female violence and other symptoms of gender-based oppression are covered in many criminology courses and textbooks.

The marginalization of critical thought is also part of a broader academic trend, and progressive insights of any sort are being pushed out of the entire field of violence studies (DeKeseredy, 2019a; DeKeseredy et al., 2023). This is not to say, though, that every 1 of the 12 distinct feminist theories (see Table 1) used to explain violence and other types have entirely disappeared.^1^ In fact, *liberal feminism* maintains hegemony in the field of violence against women and in society at large (Kiraly and Tyler, 2015a; DeKeseredy, 2021a; Pease, 2019). Liberal feminists contend that women are discriminated against based on their sex, as they are denied access to the same political, financial, career, and personal opportunities as men (Brubaker, 2019). For them, the problem of gender inequality can be solved by clearing the way for “women's rapid integration into what has been the world of men” (Ehrenreich and English, 1978, p. 19).

**Table 1:** Twelve types of feminism.

**Liberal feminism**
Radical feminism
Cultural feminism
Socialist/Marxist feminism
Intersectional feminism
Ecofeminism
Black feminism
Postcolonial feminism
Anarchist feminism
Postmodern feminism
Transfeminism
Global feminism

Liberal feminism has successfully survived because it champions individualism, does not threaten the capitalist, patriarchal status quo and reassures the public that feminists are not a “scary other” (Jovanovski, 2015; Kiraly and Tyler, 2015b). The current scenario in the field of woman abuse is arguably best described by Pease (2019, p. 5):

[L]iberal feminist ideas have gained dominance. Social movement politics against men's violence informed by radical, socialist and multicultural feminism have been supplanted by liberal feminist, public health and professionalized approaches to violence prevention. Consequently, we have witnessed a deradicalization of feminism and gender analyses, strategies for men that overemphasize reconstructing masculinity rather than challenging patriarchy, “a not all men” refrain from so-called “good men,” and a greater acceptance of anti-feminist politics within the mainstream.

On top of ignoring broader patriarchal forces and other macro-level types of inequality in their theoretical and policy work, liberal feminists are ardent advocates of what Mills (1959) refers to as *abstracted empiricism* (e.g., research divorced from theory; DeKeseredy et al., 2023). Prime examples discussed in feminist historical work on university/college campus sexual assault are Fisher et al. (2000, 2010). These researchers emerged shortly after University of California, Berkeley social work professor Gilbert (1991), one of the most well-known supporters of the backlash, sharply attacked the path-breaking national U.S. campus sexual assault survey conducted by Koss et al. (1987). He claims that the high rates of victimization discovered by Koss and her colleagues are inaccurate and the products of using broad definitions of sexual assault advanced by *radical feminists* who are mainly concerned with meeting their left-wing political goals. In Gilbert (1991, p. 61), their mission is to “impose new norms governing intimacy between the sexes.” It is unclear whether Gilbert is knowledgeable about radical feminism but leading experts in the field know that it is a school of thought that asserts that the most important set of social relations is found in patriarchy. All other social relations, like class, are secondary and originate from male-female relations (Beirne and Messerschmidt, 2014; Miller, 2021).

Gilbert (1991, p. 59) contends that some behaviors included in the four categories of sexual assault operationalized by Koss et al. are not sexual assaults. To be more precise, he points to what he defines as flaws in how Koss et al. measured rape and attempted rape, arguing that their approach “casts a large, tightly-woven net that snares the minnows with the sharks” and further that they:

Imply an understanding of sexual encounters that does not square with human attitudes and experiences. According to this view, a young woman who embraces a man who attracts her must know decisively whether or not she wants to have sexual intercourse at every given moment. Moreover, she must communicate these sentiments explicitly before any physical contact occurs. This perception does not allow for the modesty, emotional confusion, ambivalence, and vacillation that inexperienced young people feel during the initial stages of intimacy (1991: 6).

Fisher et al. (2000; 2010, p. 25) enthusiastically entered the arena. They assert that their National College Women Sexual Victimization Survey exemplifies objective, value-free science and that they “are not cultural warriors anxious to enter the fray,” but are researchers who can “help resolve the extant debates by producing solid estimates of the extent to which female students risked sexual victimization.” They also maintain that they “took the best and avoided the worst” of the methods used by the National Crime Victimization Survey and Koss et al. (1987), a claim that is not universally accepted and subject to criticism by highly seasoned experts in the field (e.g., Muehlenhard et al., 2017; DeKeseredy et al., 2023).

For Fisher et al. (2010, p. 27), “In the end, methods matter.” They do, but so do well-defined concepts. Definitions of woman abuse are important and warrant considerable scrutiny because of the power conveyed by “scientific” and “political authority” (Muehlenhard et al., 1992). The ways acts of male-to-female violence are defined have major effects on research techniques, theory construction, policies, and ultimately, the lives of many people (DeKeseredy and Schwartz, 2011). Further definitions are used politically as tools in social struggles. Together with poverty, unemployment, terrorism, and other social problems, woman abuse is a highly politicized topic of social scientific inquiry and definitions of this harm reflect this reality (Ellis, 1987).

One of the first signs of the beginning of the above situation described by Pease (2019) is the work of Fisher et al. (2000, 2010). So is the return of legalistic, *gender-neutral definitions* (DeKeseredy, 2021a). In fact, since the late 1990s, we have seen a proliferation, particularly in the U.S., of academics, government agencies, and right-wing men's groups who use narrow conceptualizations of abuse and terms like *intimate partner violence* (IPV). For reasons offered in the next section, it is time for these ways of naming the problem to go away and to revisit broad, *gender-specific* conceptualizations of woman abuse.

## The value of broad, gender-specific definitions

In the field of violence against women, there are two distinct calls for putting old wine in new bottles (DeKeseredy and Dragiewicz, 2007). One is made by groups advocating the use of gender-neutral terms like IPV, *spousal violence*, and *domestic violence*, while the other comes from radical and other critical feminists who want to rekindle the application of gender-specific terms like *violence against women* and *woman abuse*. Gender-neutral terms were common in the 1970s when scholars affiliated with the University of New Hampshire's Family Violence Research Program monopolized journal articles and scholarly book chapters on “violence behind closed doors.”^2^ Led by Straus, the New Hampshire school, based on data generated by his (1979) Conflict Tactics Scale (CTS) and later by the revised CTS (see Straus et al., 1996),^3^ erroneously claimed that women are as violent as men. Starting with the work of Breines and Gordon (1983), a large literature shows that nothing can be further from the truth. Thus, gender-specific terms like violence against women became the norm and the New Hampshire school's approach, thanks to critical feminist scholarship, became marginalized in the mid-1980s (DeKeseredy and MacLeod, 1997).

Still, fathers' rights groups and other conservative men's organizations relentlessly lobbied North American government agencies to use gender-neutral terminology in their efforts to reassert patriarchy, and they eventually won this battle in Canada and the U.S. at the start of this millennium (DeKeseredy, 2021a; DeKeseredy and Dragiewicz, 2007).^4^ For example, Statistics Canada sharply deviated from employing methods included in its national Violence Against Women Survey (VAWS) that were *women-centered* (Johnson, 1996). It administered surveys in 1999 and 2004 that were specifically crafted to produce sexually symmetrical CTS data (DeKeseredy and Schwartz, 2003).^5^

South of the border, the claim that “women do it too” was used during President George W. Bush's tenure to undermine the Violence Against Women Act (VAWA) and efforts to support it (Dragiewicz, 2011).^6^ VAWA, mainly because of the political efforts of the aforementioned men's organizations, now views women *and* men as victims of intimate violence and sexual assault, and it allows for provisions of services to men. This is a major transition because, as noted by Dragiewicz (2008, p. 130), VAWA “was passed in part because the existing ‘gender-neutral' laws were not being enforced equitably in the context of the patriarchal subordination of women. Police failure to respond to men's violence against female intimates was pervasive prior to VAWA.”

Today, a large cadre of predominantly liberal feminists have unintentionally created an unnatural partnership with patriarchal men's groups by, as Sheehy (2018, p. 251) puts it, “abandoning the language of male violence against women in favor of ‘gender-based violence' – a term so vague and depoliticized that it can signal many forms of violence, including violence against men.” Pease (2019, p. 5), and rightfully so, asserts that this language shift stems from feminist scholars and activists feeling pressured to “locate themselves in the dominant discourse to enable them to gain some traction on women's victimization. This means that they have been able to soften their analysis or omit some aspects of their understanding of the problem.” Some observers (e.g., Jordan, 2009) state that this name change reflects a change in the operationalization of violence against women and more inclusiveness regarding the types of relationships in which violence is experienced (e.g., married and non-married, current and former relationships, LGBTQ+ companionships).

Inclusivity is vital, but other ways of being unifying do not align with the interests of men's rights activists (MRAs) and other conservative, heteronormative coalitions. It is also not enough to claim that progressive inclusive politics and analyses are distinct from right-wing agendas because the reality is that “[n]eutral terminology coupled with MRAs attacks on spaces and services dedicated to women survivors have facilitated a constriction of resources dedicated to women and a blurring of who does what to whom” (Sheehy, 2018, p. 251). Therefore, to avoid supporting the goals of MRAs and the women who stand behind them, more specific terms to describe the abusive experiences of LGBTQ+ community members should be used (see DeKeseredy, 2021a). Salient examples are *intimate violence against lesbian partners, intimate violence against gay partners*, and *intimate violence against trans partners*.

In sum, Coy's (2024: 133) message warrants careful consideration:

Well-intentioned attempts to be inclusive of all victim-survivors… obscures the evidence that most perpetrators are men. Doing so throws away the opportunity to interrogate patriarchal and harmful masculinities and male peer support, including how these might be shaped by race [and] class). There are practical consequences here. Equipping women with skills and confidence has long been a feminist priority… But if the work stops there, it becomes about responsibilizing women. [I]nstead the focus should be on perpetrators: “how about telling men not to rape?”

In addition to seeing the disappearance of gender-specific definitions, we are now seeing a shift away from requiring doctoral students to write traditional dissertations. The following discussion will focus on the problems associated with this major change in academic training.

## “Tomorrow people, where is your past?”: the importance of writing a traditional Ph.D. dissertation

The above heading includes the title of a 1988 song composed and performed by Ziggy Marley, the eldest son of reggae legend Bob Marley, who died in 1981. Ziggy's song “reminds us that we must know our past to progress forward” (Songfacts, 2024, p. 1). The same can be said about academics regardless of what they study. I cannot speak for all subfields of criminology or sociology, but increasingly, we are seeing newcomers to the study of violence against women who are not mandated to develop as rich an understanding of the history of this field as novice researchers were 10 years ago (DeKeseredy et al., 2023). This is because doctoral students can now substitute the traditional dissertation with the “three-paper” option. Justified by many as beneficial for the current highly competitive job market, this route precludes the development of a broader, in-depth grasp of a subject matter because comprehensive reviews of background information are typically not essential (Arvan, 2019; European/International Joint Ph.D. in Social Representation Communication, 2022).

Developing a three-paper dissertation often entails crafting a *scoping review*, which is defined by many scholars today as “original gold standard research.” Scoping reviews are basically syntheses of how research is conducted in a certain area or field, but it is my experience and that of others (e.g., Khalil et al., 2020), that many Ph.D. students overlook or ignore important sources and only examine the most recent publications that appear in mainstream journals that deliberately omit critical scholarship (e.g., *Partner Abuse* published by Springer; DeKeseredy, 2021a; DeKeseredy et al., 2023). Further, scoping reviews are mainly exploratory/descriptive are not explanatory/analytical in nature (Khalil et al., 2020). In the words of Capozzi (2024, p. 1), unlike a meticulous literature review found in a traditional dissertation or in-depth review of the extant empirical and theoretical work in one's field, “there is no rigorous critical appraisal.” Further, there is mounting evidence that many studies cited in scoping and *systematic reviews* are fabricated and falsified (Else, 2024). Systematic reviews, too, are now in fashion, and are written to synthesize numerous studies on a specific topic and extract a broader conclusion (Capozzi, 2024).

Sadly, with the emphasis now on writing three-paper dissertations and doing scoping reviews, the academy now graduates a large cohort of early scholars who lack critical thinking skills, write papers missing deep analysis and offer little more than superficial insights (Tricco et al., 2018), and who produce atheoretical scholarship characterized by abstracted empiricism. For example, Ip et al.'s (2024) crafted a study to examine the types of theories of, and theoretically informed explanations for, sexual victimization/perpetration in higher education (colleges and universities) that were published in peer-reviewed journals from 2013 to 2022. The sample consists of 292 articles in 10 violence-related periodicals listed in the Social Sciences Citation Index, and sexual assault was the dependent variable in all the studies presented in them. Ninety-seven percent of the studies are purely quantitative, 44% are simply empirical studies with no theoretical frameworks, and 56% were informed by a theory. Not surprisingly, in this current era, Ip et al.' analyses found that of the articles guided by theories, 68% were informed by individualistic-positivistic perspectives that prioritize micro and individualistic factors, while only 30% were guided by feminist theories that give precedence to patriarchy and masculinity.

Ip et al.'s study shows that the current state of social scientific knowledge about sexual assault is haunted and possessed by a methodological conservatism (and the same can be said about the study of woman abuse in general) that is reluctant to accept alternative modes of inquiry consisting of creative and reflexive imaginations. However, as Ip et al. demonstrate, imagination is not necessarily anti-science. Albert Einstein, for instance, wrote a dissertation and published four papers based on his doctoral research. He got his initial ideas of the theory of general relativity just by daydreaming about a person falling off a building. If scholars who examine woman abuse sincerely want to grasp all the harms that exist on Kelly's (1987, 1988) ground-breaking *continuum of sexual violence* by their roots and employ truly effective strategies, there is a need to conduct rigorous, thorough reviews of the extant literature (including theoretical offerings), move beyond abstracted empiricism, apply the sociological imagination, and embrace non-positivist methodologies (DeKeseredy et al., 2023). What Stanko (2006, p. 554) declared nearly 20 years ago stands the test of time: “What is missing from a general understanding of violence is asking what can be learned from the struggles feminists have waged for decades now against physical and sexual violence.” Again, tomorrow people, where is your past?

## Revisit self-report surveys of potential male offenders

Most violence against women researchers, regardless of methodological preferences, would strongly agree with Sparks (1981, p. 49) statement about the value of victimization surveys:

The survey of victimization is only one weapon in the researcher's arsenal, of course; no single research technique can do everything. But there is no doubt that it is a potentially powerful weapon – and one that can be used to great advantage against ignorance and misconception where many problems about crime are concerned.

The victimization survey is the technique most often used to collect quantitative woman abuse data and self-report surveys of potential adult male perpetrators are in short supply (DeKeseredy, 2021b). One salient departure from this trend is Yount et al.'s (2016) survey of men's perpetration of psychological, physical, and sexual victimization of wives in rural Vietnam.

What accounts for the dearth of self-report surveys? Maybe Jaquier et al. (2011, p. 26) provides the best answer:

It is generally agreed that population surveys, in which random samples of women are interviewed about their experiences of violence using detailed, behaviorally specific questions yield more valid and reliable estimates of the prevalence of these phenomenon in the population….

Most self-report crime surveys measure delinquent activities by high school students and tend to focus on relatively minor offenses like using drugs or alcohol (DeKeseredy, 2019b). Of course, some self-report surveys have focused on the abuse of women in university/college dating and other intimate, heterosexual contexts (e.g., Straus and Gelles, 1986; DeKeseredy and Schwartz, 1998), but it is fair to claim that one of the largest gaps in our social scientific knowledge about male-to-female violence remains self-reported adult male abusive behaviors.

Because of social desirability effects and other factors, listening to women's voices and inviting them to complete victimization surveys enables researchers to uncover much higher estimates of any type of woman abuse than those derived from self-report surveys administered to men (DeKeseredy, 2019b). Still, the research community is at the point where it can confidently state that a substantial number of women experience male violence. Hence, it is now time to revisit self-report survey technology used decades ago by some woman abuse researchers like those cited in the previous paragraph to yield better answers to some very important questions, such as “Why Does He Do That?” (Bancroft, 2002). As the late Scully (1990, p. 4) observes, there are problems in completely depending on females to report male abusers because “Women cannot reveal the motivations and justifications of the men who [abuse] them because they don't share the reality of… violent men. Such insight is acquired through invading and critically examining the social constructions of… men.”

## Quantitative indicators of men's patriarchal attitudes and beliefs

In the introduction to their anthology *Violence Against Women*, Renzetti and Kennedy Bergen (2005, p. 5) direct readers to one of Diana Scully's most important contributions to a feminist understanding of rape. She and Joseph Marolla (see Scully and Marolla, 1985) interviewed 100 convicted rapists and, as described by Renzetti and Kennedy Bergen, “Regardless of their motivations, Scully and Marolla show that the rapists shared a sense of entitlement to women, whom they objectified, and they enjoyed the dominance over women that rape gave them.” Thus, some feminists like Dragiewicz (2009, p. 204) argue that “It is impossible to have an adequate discussion of sex, gender, and violence without talking about patriarchy.” Nonetheless, as made explicit by Ptacek (2023, p. 14), among others (e.g., DeKeseredy, 2021a), “In feminist writing, the term ‘patriarchy' has apparently fallen out of use in recent years,” and it is now time to resurrect this concept and gather quantitative data on abusive men's patriarchal attitudes and beliefs as was done by some Canadian feminist survey researchers in the early 1990s.

For example, modified versions of definitions of *familial patriarchy* offered by Smith (1990) and DeKeseredy and Schwartz (1993) informed one used in DeKeseredy and Schwartz's (1998) Canadian National Survey of Woman Abuse in University/College Dating (CNS): *a discourse that supports the abuse of women who violate the ideals of male power and control over women in intimate relationships*. Relevant themes of this ideology are an insistence on women's obedience, respect, loyalty, dependency, sexual access, and sexual fidelity (Dobash and Dobash, 1999; Barrett and MacIntosh, 1982; Pateman, 1988).

These themes were operationalized with two indices used by Smith (1990). One index measures patriarchal beliefs, and the other measures patriarchal attitudes. Cronbach's alpha coefficients (0.79 for beliefs and 0.76 for attitudes) show that these indicators are reliable. Further, DeKeseredy and Schwartz found that college/university men who espouse patriarchal attitudes and beliefs were more likely to engage in sexual, physical, and psychological abuse than those who do not report abusive behavior. These men were even more likely to abuse their female dating partners if they were influenced by *male peer support*, which is attachments to male peers and the resources that these men provide that encourage and legitimate woman abuse (DeKeseredy, 1988).

Some scholars (e.g., Oakley, 2000) contend that only qualitative work that examines patriarchy constitutes pure or inherently feminist research. There are also those who declare that feminist empirical, theoretical, and policy concerns are incompatible with the goals of mainstream social science. The quantitative indicators of patriarchy used by Smith (1990, 1994) and DeKeseredy and Schwartz (1998) demonstrate that nothing is further from the truth. Over the past 40 years, we have witnessed significant advances in victimization and self-report survey research thanks to the efforts of feminist scholars. Moreover, many feminists today recognize that their empirical concerns can be effectively addressed by adhering to the rules of orthodox social science (DeKeseredy, 2016). For example, over the past 30 years, I and some of my colleagues have conducted several surveys that involved using advanced statistical techniques and several feminist qualitative methods based on the success of Smith (1987; 1990; 1994) Toronto woman abuse survey, such as multiple measures of abuse and supplementary open- and closed-ended questions. The benefits of these two techniques are covered in the next two sections of this article.

## Supplementary open-ended survey questions

Even with all the methodological advances that have occurred over the past 50 years, “Obtaining accurate estimates of the extent of woman abuse… remains perhaps the biggest methodological challenge in survey research on this topic” (Smith, 1987, p. 185). There are a wide variety of reasons that both abused women and male offenders might not disclose incidents. These include embarrassment, fear of reprisal, *forward and backward telescoping*, deception, and memory error (DeKeseredy and Schwartz, 2013; DeKeseredy et al., 2021). Others suggest that underreporting can come from the reluctance to recall traumatic incidents and the belief that violent or psychological assaults are too trivial or inconsequential to mention (Smith, 1994; DeKeseredy and Schwartz, 1998).

These problems are difficult to overcome and not likely to be eliminated soon, if ever. Even so, there are effective ways of minimizing underreporting, and one is to add supplementary open-ended questions to quantitative surveys. Yet, many, if not most, violence against women surveys disregard this recommendation and only use modified versions of the CTS or Koss et al.'s (2007) Revised Sexual Experiences Survey (RSES). These are reliable and valid measures (DeKeseredy et al., 2021; Krahe, 2024), and they are widely used in Canada, the U.S., and elsewhere. Nevertheless, they ignore many injurious acts like stalking, suffocation, and many types of technology-facilitated violence against women (DeKeseredy, 2019b).

Another problem with only using the CTS or RSES is that respondents are not given additional opportunities to disclose abusive experiences. At the outset, people may not report incidents for reasons described previously (e.g., embarrassment, fear of reprisal). However, some large-scale surveys show that if respondents are probed later by an interviewer or asked to complete supplementary open-ended questions, some silent or forgetful participants will reveal having been victimized (DeKeseredy et al., 2019). Further, these strategies provide “deep insights into participants thinking and behavior” (Conry-Murray et al., 2024, p. 15). For example, Smith (1987) found that sixty female victims of male violence who participated in his Toronto woman abuse survey and who did not initially reveal their experiences changed their answers when asked again in different words by a telephone interviewer. Belated responses to his three supplementary open-ended questions increased the overall physical violence *prevalence* rate from 25% to 34%, and 29 belated disclosures increased the severe violence prevalence rate from roughly seven percent to nearly 12%. Smith defined prevalence as the percentage of women who ever reported having been physically abused.

On top of giving respondents more opportunities to disclose abusive events, supplementary open-ended questions like this modified version of Smith's (1987) used in the Campus Quality of Life Survey (CQLS) conducted by DeKeseredy (2019b, 2021) and Pritchard et al. (2019) help build researcher-respondent rapport:

We really appreciate the time you have taken to complete this survey. And, we'd like to assure you that everything you told us will remain *strictly anonymous*.

We realize the topics covered in this survey are sensitive and that many students are reluctant to talk about their own campus experiences. But we're also a bit worried that we haven't asked the right questions.

So now that you have had a chance to think about the topics covered in this survey, would you like to provide us with any additional information about the quality of life on this campus? If so, please use the box below.

Like the rest of your responses to this survey, any information you provide is anonymous and will only be reported grouped with other comments.

Of the total CQLS sample (*n* = 5,718) who enrolled at an institution based in the South Atlantic part of the U.S., 3,271 were women (including both undergraduate and graduate students) and a total of 410 of these female respondents (13%) answered the above question. Twenty-six of them (0.79%) provided belated reports of victimization that were not gleaned by the quantitative measures. In the words of Smith (1987, p. 182), “they either had second thoughts about their prior decision not to disclose their experience or remembered a previously forgotten incident.” Another possibility raised by Smith is that some abuse measures do not describe some people's particular events. This respondent provides examples of behaviors reported in the qualitative section of the CQLS instrument that increased the rate of victimization:

Freshman year I was at a bar downtown and a guy was trying to talk to me and I was clearly ignoring him. Then he forcefully grabbed me by the back of the neck and forced me to kiss him. Some other males intervened and got him kicked out. After they came up to me and said they had been watching him because he raped a girl the weekend before. It was well known what he had done, yet he was still downtown hunting for his next victim. I feel a HUGE issue with males on this campus is their sense of entitlement to the females' bodies. I have lost count of the times I have had my butt grabbed by males I have never even spoken to. Catcalling is huge downtown – I have had a man whisper in my ear he was going to rape me and had countless comments telling me to smile, that I looked good or about my boobs bouncing as I walked. I have had strangers make jokes to me saying that they know me and when I said I didn't recall they said it was because they drugged me (quoted in DeKeseredy et al., 2021, p. 2484-2485).

Three-hundred and twenty-one (78.2%) of the four-hundred and ten women who answered the above question did not reveal any experiences of the four types of woman abuse examined in this study,^7^ but instead centered on problematic university campus responses to violence against women, racism, and homophobia. Consider this woman's response:

The university condones significant drinking and the drug scene is escalating. Punishments are not harsh enough for sexual offenders and there is still a huge stigma against women. I taught a student who was assaulted and I was appalled that someone asked me, “Well what was she wearing? Why was she alone?” The campus could be much safer but it needs to be a major culture shift. I have also heard many (mainly White) students make inappropriate comments about sexism, and homophobia appears to have become worse in several years (quoted in DeKeseredy et al., 2021, p. 2486).

Though it was a victimization survey, the CQLS, with the help of the open-ended question, also yielded some information from men about their sexist and racist attitudes and beliefs. For instance, DeKeseredy et al. (2019) found male narratives that (a) deny high rates of female sexual victimization and make claims of false accusations and (b) that reflect anger at, or disdain for, women, ethnic and sexual minorities, and campus, diversity, equity, and inclusion policies.

One of the main strategies that males referred to by Kimmel (2017) as *angry white men* repeatedly used to deal with “their problems” is claim that they are the “real victims” and that rates of male-to-female sexual assault in institutions of higher learning are greatly exaggerated (DeKeseredy et al., 2015). This is one of the key themes found in the CQLS qualitative data. A white, heterosexual, male undergraduate vividly highlights a variation of this theme which DeKeseredy et al. (2019, p. 9) found in 18 narratives:

This survey will undoubtedly be used to show that White men are bad and don't have any of the bad experiences that “people of color” or refrigerator gendered people face… Furthermore, the school police should not handle sexual assault cases. This is to be handled by actual law enforcement not connected to the school. That way there isn't any bias. If one did occur and the perpetrator is found guilty, the school should take whatever action it deems necessary. But we live in a country of innocent until proven guilty. The university should at least act like it. But, because I have checked “White” and “male” in your survey, none of this matters to you.

Angry white men, including those who are college/university students, blame members of LGBTQ+ and/or specific ethnic communities for their “problems” (Kimmel, 2017). Eleven male CQLS narratives illustrate this theme, and this is one prime example:

All I can say is kids on this campus need to stop being pussies and stick up for themselves instead of tattling. Snitches deserve whatever they get for being babies. This school is sheltering these kids too much and it's wasting the resources. If they can't handle living at college and getting a taste of what the world is like, they should go home to suckle mommy and daddy's teats. These “special snowflakes” should receive no special accommodations and should accept and prepare for the harassment and ridicule that comes with rainbow hair or the flaunting how gay you are (quoted in DeKeseredy et al., 2019, p. 12).

Distinguished Black scholar West (2001, p. 4) points us to the fact that “race matters.” He also notes, “our truncated public discussions of race… fail to confront the complexity of the issue in a candid and critical manner.” Race also matters to some male CQLS respondents, but the discussion they would like is profoundly different from that sought by West. Contemplate this student's view of his school:

This university is becoming increasingly discriminatory to people who do not fit into the category of “minority.” Straight, White, Christians are very frequently silenced in conversations about any type of social issues, especially men. This university fosters a hostile environment to those who hold conservative principles and are often told their worldview is wrong or bigoted even in classrooms (quoted in DeKeseredy et al., 2019, p. 12-13).

This White male student offers a similar observation in his response to the above supplementary question:

I have found with programs and studies like these that often times the obvious is left unspoken, such as how are the groups just talked about are racist toward other groups. I have found several ethnic backgrounds to be more racist and more likely to be loud and use the race card without hesitation in almost any circumstance regardless of the actual situation or happenings… Just because some populations, such as White men, are not constantly begging for attention, their situations are often dismissed. I would like to see more studies on this matter (quoted in DeKeseredy et al., 2019, p. 13).

In sum, though not widely used, supplementary open-ended questions like those pioneered by Smith (1987) help minimize underreporting and provide rich contextual and other types of information that cannot be gleaned quantitatively. For instance, the CQLS provided rich qualitative data on angry white men and the antifeminist backlash that the quantitative questions were not specifically designed to measure. Returning to the issue of underreporting, attempts to minimize the key sources identified previously are necessary because the negative consequences are significant. Indeed, an entire survey can be discredited if researchers cannot discern if the women who report having been abused are representative of all survivors in the sample. Struggles for effective social support services are also hindered because high levels of underreporting result in lower estimates of abuse and ultimately decrease the probability of mobilizing resources to curb female victimization (Smith, 1994; DeKeseredy, 1995, 2016).

It is naïve to assume that accurate statistics derived from large-scale representative sample surveys generally motivate state agencies to devote more resources developing effective prevention and control strategies. Then again, feminist scholars and activists like Bart et al. (1989, p. 433) fully recognize that “The principal questions that organize policy efforts are ultimately quantitative – how many are there, who are they, where are they, how bad are the consequences, how much will it cost.” Of course, methodological decisions should not be based entirely on state agencies' concerns; even so, accurate data can help influence government officials to consider implementing progressive policies (DeKeseredy, 2017).

## Conclusion

Some readers may interpret this article as little more than a nostalgic trip down memory lane or a weak attempt to repackage old ideas. Actually, the conceptual, theoretical, and empirical approaches to understanding woman abuse promoted here are proven to be useful and should not be abandoned. They have been used repeatedly and are proven to be reliable, effective, and trustworthy. From a critical criminological standpoint, above all, they facilitate the development of rigorous research that offers “the counter-voice” to abstract empiricist work that individualizes one of the world's most compelling social problems (Young, 2011).

The imaginative feminist contributions advanced here are not exhaustive; undoubtedly, many readers can probably think of more that were used in the past and continue to be used today. Undoubtedly, too, new ways of studying woman abuse will be needed because this harm is a “never-ending and constantly evolving issue” (Ledwitz-Rigby, 1993, p. 93). Ponder the “dark side” of new technologies. Leading experts on violence against women only started to address this problem in the last few years. As Goodmark (2011, p. 195) notes, “When the first domestic and stalking laws were passed, no one could have foreseen how technology would facilitate abuse, stalking, and harassment.” New technological developments, such as artificial intelligence, will facilitate various types of violence against women and progressive scholars will have to modify their research accordingly.

Still, it is always necessary to keep “looking backward to move forward,” which entails repeatedly revisiting past feminist definitional, empirical, and theoretical offerings (DeKeseredy, 2016), such as those featured in this article. Perhaps, then, it is most appropriate to conclude this piece with these two highly seasoned survey researchers' sage advice:

Amidst all of our speculation on the future – some of which will surely prove misguided – we [should] by no means lose sight of the value of continuity. We imagine that innovations… will serve to expand the range of options for survey researchers rather than to fully supplant those currently practiced… Future investigators should judiciously balance them together with the new approaches that will inevitably emerge (Wright and Marsden, 2010, p. 24).

## Footnotes

^1^Renzetti (2018) and DeKeseredy (2022) for reviews of these feminist theories.

^2^Behind closed doors is also the title of Straus' et al. (1981) widely read and cited book, which is a monograph that heavily promotes the use of gender-neutral terminology.

^3^See DeKeseredy and Schwartz (1998) for an in-depth critique of both renditions of the CTS.

^4^However, DeKeseredy and MacLeod (1997) provide evidence that in the mid-1990s, the naming of woman abuse and violence against women once again began to shift toward gender-neutral terms.

^5^See DeKeseredy and Schwartz (2003) and Dragiewicz and DeKeseredy (2012) for more information on these surveys.

^6^VAWA has been reauthorized four times since its enactment in 1994 (2000, 2005, 2013, and 2022).

^7^The CQLS included quantitative measures of intimate partner violence, sexual assault, stalking, and stranger sexual harassment.
